# A Descriptive Catalogue of the Anatomical Museum of the Boston Society for Medical Improvement

**Published:** 1848-06

**Authors:** 


*A Descriptive Catalogue of the Anatomical Museum of the Boston Society for Medical Improvement.* By J. B. S. Jackson, M. D., Curator of the Museum; Professor of Pathological Anatomy in Harvard University. 8vo. pp. 352. Boston, 1847.

The Boston Society for Medical Improvement, in many of its features, resembles the Philadelphia College of Physicians. From the Introductory Chapter to the present volume, we learn that it "was founded in the year 1828, and has continued its meetings regularly, twice a month, to the present time. At these meetings an original paper is usually read by one of the members in turn; and each one present is called upon to communicate, extemporaneously or in writing, any information or cases of interest that may have occurred to him during the intervals of the meetings, or any case concerning which he may desire to have the opinion of the society; each member thus obtaining direct information in regard to most of the important cases that have occurred in the city or vicinity."

The reports of cases made by the members are frequently accompanied with the diseased parts, obtained from post mortem examination; and it is from a selection of the specimens of morbid anatomy thus exhibited, that "the cabinet of the society has been mainly formed, and its peculiar and principal interest is due to the connection of the specimens, in most instances, with authentic histories of the cases from which they were derived." In the Catalogue, these histories are concisely given as far as they are known. The number of the specimens enumerated is nine hundred and fifty-four. The volume also contains a very copious and well arranged index, beside several excellent engravings, illustrative of various instances of monstrosity.

We can hardly use language too strong to express our admiration of the industry and liberality which has created such a museum in our northern metropolis, and of the labour and ability displayed in the preparation of its very complete catalogue.

